# Safety of fertility preservation in breast cancer patients in a register-based matched cohort study

**DOI:** 10.1007/s10549-017-4555-3

**Published:** 2017-11-02

**Authors:** Kenny A. Rodriguez-Wallberg, Sandra Eloranta, Kamilla Krawiec, Agneta Lissmats, Jonas Bergh, Annelie Liljegren

**Affiliations:** 1 Department of Oncology-Pathology, Karolinska Institutet, Stockholm, Sweden; 2 Department of Obstetrics and Gynecology, Section of Reproductive Medicine, Karolinska University Hospital, Stockholm, Sweden; 3 Regional Cancer Center, Stockholm-Gotland, Stockholm, Sweden; 4 Department of Oncology Radiumhemmet, Karolinska University Hospital, Stockholm, Sweden; 5 Reproductive Medicine Karolinska, Karolinska University Hospital Huddinge, Novumhuset Plan 4, 141 86 Stockholm, Sweden

**Keywords:** Breast cancer, Population-based register study, Fertility preservation, Relapse rate, Young age, Hormonal stimulation

## Abstract

**Purpose:**

To determine if women with breast cancer that undergo fertility preservation (FP), with or without hormonal stimulation, present with an increased risk of breast cancer recurrence.

**Methods:**

A matched cohort study on women with breast cancer attempting to ensure FP in Stockholm from 1999 to 2013 [exposed women (*n* = 188), age-matched unexposed controls (*n* = 378)] was designed using the Stockholm regional data from the Swedish National Breast Cancer Quality Register. Breast cancer relapse rates [incidence rate ratio (IRR)] and 95% confidence interval (CI) were estimated using Cox regression and adjusted for potential confounding factors. Completeness of the registry at the time of the study was close to 99%.

**Results:**

Most women attempted FP by hormonal stimulation treatment (*n* = 148, 79%) with the objective of freezing their eggs or embryos. A smaller group elected FP methods without hormone stimulation (*n* = 40, 21%). Women who received hormone stimulation did not present with a higher relapse rate than unexposed control women in a model adjusted for age and calendar period of diagnosis (IRR 0.59, 95% CI 0.34–1.04). The results remained virtually unchanged after adjustment for tumor size, estrogen receptor status, affected lymph nodes, and chemotherapy treatment (IRR 0.66, 95% CI 0.37–1.17).

**Conclusion:**

Evidence was not found that fertility preservation, with or without hormonal stimulation, was associated with an increased risk of breast cancer recurrence. The high coverage rate of this population-based study supports the safe practice of fertility preservation in young women with breast cancer.

## Introduction

Breast cancer is the most common malignancy in women and usually presents with biologically aggressive features when presenting at a young age. Hence, chemotherapy is the current standard of care for most young patients but is associated with a high likelihood of inducing ovarian toxicity and infertility after treatment [1, 2].

Infertility induced by cancer treatment is a recognized survivor issue and the practice of fertility preservation is spreading rapidly [3–5]. Currently established clinical methods of female fertility preservation include the cryopreservation of embryos and mature oocytes obtained after hormonal stimulation as the ability to thaw and use them successfully in fertility treatments has been demonstrated [4, 6]. However, owing to the biology of breast cancer, concerns have been articulated as hormonal treatment is regarded as being potentially dangerous. There is also the option of ovarian tissue retrieval which does not require hormonal stimulation. Although live births have been reported following the re-transplantation of ovarian tissue, clinical standards have not yet been set and this option is still considered to be experimental [4].

The main reason for questioning the safety of hormonal stimulation in breast cancer patients is the very high level of circulating estradiol that results from the simultaneous development of multiple ovarian follicles [7]. To overcome fears of hormonal stimulation, less effective treatments have been proposed, such as the retrieval of oocytes in the natural cycle without hormonal stimulation [8, 9] and the cryopreservation of ovarian tissue [4]. However, selected women with estrogen-negative breast cancer have undergone hormonal stimulation using standard gonadotropin-stimulation protocols for the purposes of fertility preservation.

In recent years, potentially safer stimulation protocols have been developed and have involved the addition of tamoxifen or aromatase inhibitors to existing standard gonadotropin-stimulation protocols [10, 11]. As greater efficacy has been reported following treatment with letrozole than that with tamoxifen, the former is preferred and has been used in the USA for several years [11–13].

The short-term follow-up of 79 women who elected to undergo letrozole stimulation for fertility preservation [14] and the following investigation of 120 women for a longer period indicate that this stimulation protocol may not have had a substantial impact on cancer recurrence, and in particular if lymph node involvement was absent [15]. However, the overall safety of these procedures is still not known owing to the lack of comparable groups. Additionally, data on the safety of fertility preservation through the use of standard hormonal stimulation protocols or without hormonal stimulation have not been reported for women with breast cancer.

In Sweden, cancer care and fertility preservation, indicated for medical reasons, are practiced within a public tax-funded healthcare system, whereby equal access by all citizens to health care is ensured. Swedish citizens are also provided with a 12-digit unique identity number that permits the use of national healthcare registers with prospectively collected information gathered on the entire population. The fertility preservation program at Karolinska University Hospital in Stockholm is the largest in the country, serving the entire region of Stockholm–Gotland (with a population of 2.2 million). In general, standard protocols for ovarian stimulation were applied to women without estrogen-sensitive cancer until 2010, when a new protocol was introduced that advocated the use of letrozole, in conjunction with that of gonadotropins [16]. Additional methods, such as egg retrieval in the natural cycle or cryopreservation of the ovarian tissue, are also available and are offered to breast cancer patients and, in particular, to women with estrogen-sensitive disease [17]. A healthcare program for breast cancer has existed in Stockholm–Gotland since the 1970s. Standardized therapy recommendations are provided by the Swedish Breast Cancer Group and have been updated for the past 15 years [17].

Most young women needing to undergo cancer treatment with the associated risk of infertility express a desire to have children in the future, regardless of their diagnosis, prognosis, or treatment [18–21]. The objective of our study was to investigate the long-term safety of fertility preservation practiced by women with breast cancer over the years using any of the several available options. As all cancer cases are mandatorily registered in the Swedish Cancer Registry, we designed a matched cohort study that would allow us to compare the incidence of breast cancer relapse within a cohort of women who underwent fertility preservation in Stockholm–Gotland with that of an age-matched control cohort, identified using the Swedish National Quality Register for Breast Cancer in the corresponding healthcare region.

## Methods

### Data source and patient population

The current study was designed as a matched cohort study. Women with breast cancer in whom fertility preservation had been performed between January 1999 and December 2013 (irrespective of whether or not hormonal stimulation was required) were considered to be exposed (*n* = 189). Thereafter, the exposed cohort was identified within the Stockholm Breast Cancer Registry (SBCR) by data linkage using each individual’s unique number. For all exposed women, two women who had not undergone fertility preservation, matched on age at diagnosis, were identified within the same calendar period in the SBCR. The SBCR is a population-based register which contains information on tumor characteristics, treatment, and relapse occurrence in patients diagnosed with invasive breast cancer since 1976. When missing from the register, data were obtained by reviewing the clinical medical records. The completeness of the SBCR for women diagnosed with breast cancer at age ≤ 45 years was 95% prior to 2008 and 99% for the later years.

### Ethics approval

Approval from the Regional Ethics Committee in Stockholm, Sweden (Dnr 2011/1758–31/2 and Dnr 2014/1825–32) was obtained prior to the study.

### Outcomes

The primary end-point in this study was the incidence of breast cancer relapse. The matched cohort was followed from the date of diagnosis until the first recorded relapse date and censored at death or January 1, 2015, whichever came first.

### Statistical analysis

Cox regression was used to estimate the rate of relapse for women exposed to fertility preservation relative to that for the unexposed women. Exposed women were classified according to separate exposure categories and depending on whether or not hormonal stimulation was required with the use of the fertility preservation method.

The results are presented as information on the relapse incidence rate ratio (IRR) and 95% confidence interval (CI). All fitted regression models were adjusted for the matching variable, age at diagnosis, using a restricted cubic spline function (with 4 degrees of freedom and knots placed at the minimum, maximum, 33th, and 66th centile of the distribution of age). Adjustments for potential confounders were made sequentially for the periods of diagnosis (1998–2002, 2003–2007, and 2008–2013), tumor size (T0, TIS, T1, T2, and T3), number of involved lymph nodes (0, 1–3, and > 3), estrogen receptor status (negative or positive), and whether or not chemotherapy treatment was given (“no” or “yes”). All variables selected for the regression models were chosen prior to data collection, based on their potential relevance as confounding factors in the association between the principal exposure and the primary end-point. As such, the model specifications were determined a priori and variables were not included in the models, solely based on the observed level of statistical significance.

The proportional hazards assumption was determined by the application of the Grambsch–Therneau test to the Schoenfeld residuals obtained from each model [22]. A two-sided test was used to determine if the results were statistically significant at the 5% level. The effects of confounders that were found to be non-proportional were managed by stratifying the Cox model according to the confounder in question. In addition, unadjusted Kaplan–Meier estimates and model-based predictions of relapse-free survival were estimated for each of the three exposure categories. The adjusted survival curves were approximated using a flexible parametric survival model adjusted for the same potential confounding factors as those in the fully adjusted Cox regression model [23]. Cases with incomplete data were excluded from Cox regression analysis to facilitate a comparison between the models. Stata^®^ 13 (Stata Statistical Software: Release 13, College Station, TX: StataCorp LP) was used for the statistical analysis.

## Results

### Study population characteristics

**Fig. 1:**
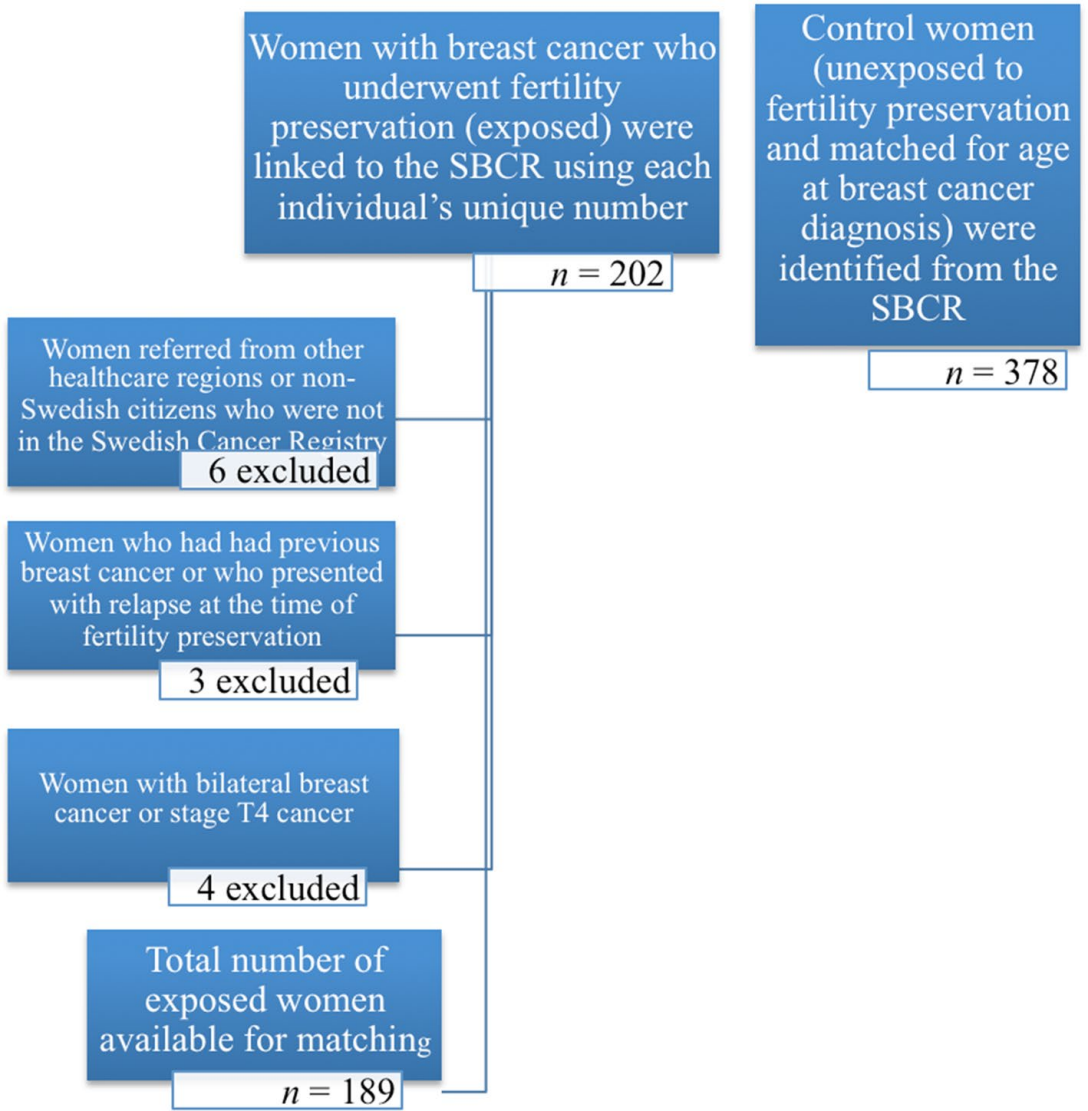
A flowchart of women with breast cancer who were included in the matched cohort study. Women exposed to fertility preservation underwent treatment between 1999 and 2013. For all exposed women, two women matched for age at diagnosis who had not undergone fertility preservation (unexposed) were identified using the Stockholm Breast Cancer Registry. SBCR: Stockholm Breast Cancer Registry

Five hundred and sixty-seven women were included in the study, of whom 189 were exposed to a fertility preservation method. There were 378 age-matched controls (Fig. 1). After matching, one exposed women was excluded owing to the absence of information. Nevertheless, the two matched controls were retained in the control cohort. A higher proportion of women in the exposed cohort underwent fertility preservation with hormonal stimulation aiming at freezing their eggs or embryos (79%, *n* = 148), whereas only 21% chose to freeze their ovarian tissue or attempted egg retrieval without hormonal stimulation (*n* = 40). The clinical characteristics of the study cohort are presented in Table 1. A high percentage of the women received chemotherapy as adjuvant treatment because of their young age at breast cancer diagnosis, in concordance with international guidelines [1]. The mean follow-up time was 6.6 years (a range of 0.3–17.9 years) and the median was 5.8 years. Very short follow-up occurred for some of the cases when the women experienced an early relapse. Of the women who were diagnosed with breast cancer in 2010 or later (*n* = 165), 78 underwent fertility preservation for which hormonal stimulation was required. Of these, 36 women (46%) were treated using letrozole. Eight relapses occurred in the cohort diagnosed with breast cancer after 2009. Six of these were women who did not undergo fertility preservation. One patient received stimulation using letrozole and another received standard ovarian stimulation.

**Table 1:** A description of the demographic and tumor characteristics in the matched cohort

Demographic and tumor characteristics	Women exposed to fertility preservation requiring hormonal stimulation		Women exposed to fertility preservation with no need for hormonal stimulation		Matched comparators (unexposed to fertility preservation)	
	*n*	Relapses (%)	*n*	Relapses (%)	*n*	Relapses (%)
Total	148	16 (10.8)	40	9 (22.5)	378	104 (27.5)
Age at diagnosis (years)						
Mean (range)	32.7 (21–42)	–	32.0 (23–38)	–	34.1 (23–42)	–
Year of diagnosis						
1997–2002 2003–2007 2008–2013	12 (8.1) 29 (19.6) 107 (72.3)	4 (33.3) 6 (20.7) 6 (5.6)	9 (22.5) 18 (45.0) 13 (32.5)	3 (33.3) 4 (22.2) 2 (15.4)	118 (31.2) 125 (33.1) 135 (35.7)	60 (50.1) 31 (24.8) 13 (9.6)
Tumor size						
T0 TIS I (≤ 20 mm) II (21–50 mm) III (≥ 50 mm) TX	5 (3.4) 1 (0.7) 79 (53.4) 55 (37.2) 17 (4.7) 1 (0.7)	0 (0.0) 1 (100.0) 6 (7.6) 9 (16.4) 0 (0.0) 0 (0.0)	1 (2.5) 1 (2.5) 20 (50.0) 12 (30.0) 4 (10.0) 2 (5.0)	0 (0.0) 0 (0.0) 5 (25.0) 3 (25.0) 1 (25.0) 0 (0.0)	25 (6.6) 1 (0.3) 158 (41.8) 144 (38.1) 46 (12.2) 4 (1.1)	1 (4.0) 0 (0.0) 41 (26.0) 47 (32.6) 15 (32.6) 0 (0.0)
Lymph nodes						
0 1–3 > 3 Missing	95 (64.2) 40 (27.0) 12 (8.1) 1 (0.7)	8 (8.4) 5 (12.5) 3 (25.0) 0 (0.0)	20 (50.0) 12 (30.0) 7 (17.5) 1 (2.5)	4 (20.0) 3 (25.0) 2 (28.6) 0 (0.0)	204 (54.0) 104 (27.5) 52 (13.8) 18 (4.8)	41 (20.1) 32 (30.1) 27 (51.9) 4 (22.2)
Receptors						
ER+ ER− ER missing	103 (69.6) 44 (29.7) 1 (0.7)	11 (11.0) 4 (9.3) 1 (100.0)	25 (62.5) 14 (35.0) 1 (2.5)	5 (20.0) 4 (28.6) 0 (0.0)	243 (64.3) 125 (33.1) 10 (2.7)	67 (27.6) 35 (28.0) 2 (20.0)
Laterality						
Right Left Missing	67 (45.3) 79 (53.4) 2 (1.4)	4 (6.0) 12 (15.2) 0 (0.0)	15 (37.5) 24 (60.0) 1 (2.5)	3 (20.0) 6 (25.0) 0 (0.0)	188 (49.7) 190 (50.3) 0 (0.0)	55 (29.3) 49 (25.8) 0 (0.0)
Neoadjuvant treatment						
No Yes Missing	123 (83.1) 23 (15.5) 2 (1.4)	15 (12.2) 1 (4.4) 0 (0.0)	29 (72.5) 10 (25.0) 1 (2.5)	7 (24.1) 2 (20.0) 0 (0.0)	291 (77.0) 87 (23.0) 0 (0.0)	70 (24.1) 34 (39.1) 0 (0.0)
Chemotherapy						
No Yes	19 (12.8) 129 (87.2)	2 (10.5) 14 (10.9)	5 (12.5) 35 (87.5)	2 (40.0) 7 (20.0)	98 (25.9) 280 (74.1)	25 (25.5) 79 (28.2)

*ER* estrogen receptor

Of the remaining 566 women included in the study, 534 (94%) were included in the Cox regression model. Of these, 351 women were matched controls, and 145 and 38 women received fertility preservation with and without hormonal stimulation, respectively. A small proportion, 6% of all the women, but only 3% of the exposed women, were not included in the analysis owing to incomplete data.

### Overall outcomes

There was no evidence of a statistically significant effect for fertility preservation effect with or without hormonal stimulation on the rate of breast cancer relapse after adjustment for age at diagnosis, period of diagnosis, tumor size, estrogen receptor status, affected lymph nodes, and chemotherapy treatment (IRR 0.66, 95% CI 0.37–1.17 and IRR 0.83, 95% CI 0.42–1.67), respectively) (Table 2). The rate of relapse was higher in women who presented with tumor sizes ≥ 20 mm (IRR for TII of 21–50 mm: 1.53, 95% CI 1.03–2.29) than that in those with tumors < 20 mm (IRR for TIII of 50 mm: 1.42, 95% CI 0.77–2.60). Relapse rate was also higher in women with lymph node involvement (IRR for 1–3 lymph nodes: 2.00, 95% CI 1.26–3.18, IRR for > 3 lymph nodes: 2.91, 95% CI 1.73–4.90). Statistical evidence was not found in the fully adjusted model in favor of the proportional hazards assumption that the presence of a greater number of involved lymph nodes would have a detrimental effect that was statistically significant. However, when the model was controlled for the non-proportional effect of the number of involved lymph nodes using stratified Cox regression, the effect of fertility preservation with hormonal stimulation remained virtually unaltered (IRR: 0.65, 95% CI 0.37–1.16).

**Table 2:** A comparison of the incidence of relapse in exposed women with breast cancer who had undergone fertility preservation and that in women who had not been exposed to fertility preservation (*n* = 534)

	Model 1^a^ IRR (95% CI)	Model 2^b^ IRR (95% CI)	Model 3^c^ IRR (95% CI)	Model 4^d^ IRR (95% CI)
Fertility preservation				
No fertility preservation Fertility preservation that does not require hormonal stimulation Fertility preservation that requires hormonal stimulation	1.00 (reference)^e^ 0.80 (0.40–1.60) 0.59 (0.34–1.04)	1.00 (reference)^e^ 0.82 (0.41–1.64) 0.65 (0.37–1.15)	1.00 (reference)^e^ 0.75 (0.37–1.50) 0.67 (0.38–1.19)	1.00 (reference)^e^ 0.83 (0.42–1.67) 0.66 (0.37–1.17)
Period of diagnosis				
1997–2002 2003–2007 2008–2013	1.00 (reference) 0.50 (0.33–0.76) 0.32 (0.18–0.54)	1.00 (reference) 0.53 (0.35–0.80) 0.33 (0.19–0.56)	1.00 (reference) 0.52 (0.34–0.79) 0.30 (0.17–0.53)	1.00 (reference) 0.52 (0.35–0.79) 0.34 (0.19–0.58)
Tumor size				
T0 TIS I (≤ 20 mm) II (21–50 mm) III (> 50 mm)		0.20 (0.03–1.47) – 1.00 (reference) 1.47 (1.00–2.17) 1.34 (0.74–2.42)	0.20 (0.03–1.47) – 1.00 (reference) 1.22 (0.79–1.87) 0.86 (0.43–1.73)	0.19 (0.03–1.40) – 1.00 (reference) 1.53 (1.03–2.29) 1.42 (0.77–2.60)
Lymph nodes				
0 1–3 > 3		1.00 (reference) 1.73 (1.14–2.62) 2.45 (1.55–3.87)	1.00 (reference) 1.71 (1.12–2.60) 2.25 (1.40–3.62)	1.00 (reference) 2.00 (1.26–3.18) 2.91 (1.73–4.90)
Receptors				
ER+ ER−			1.00 (reference) 1.01 (0.68–1.49)	1.00 (reference) 1.12 (0.75–1.69)
Neoadjuvant treatment				
No Yes			1.00 (Reference) 1.85 (1.13–3.04)	–
Chemotherapy				
No Yes				1.00 (reference) 0.64 (0.37–1.12)

^*CI* confidence interval, *IRR* incidence rate ratio, *ER* estrogen receptor^

^a^Adjusted for age at diagnosis (using a restricted cubic spline with 4 degrees of freedom) and the calendar period of the diagnosis

^b^Further adjusted for tumor size and the number of involved lymph nodes

^c^Further adjusted for estrogen receptor status and neoadjuvant treatment

^d^Adjusted for chemotherapy treatment (pre- or postoperative)

^e^Evidence for the proportional hazard assumption

In Sweden, Her2 screening was initiated in August 2005 and adjuvant trastuzumab treatment recommended. To determine whether Her2 status would have impacted on our estimates, further reduced time spans in the calender period designated for the matching of the cases and controls are presented (Table 3). The IRR for fertility preservation treatment remained robust after the adjustments.

**Table 3:** A comparison of the incidence of relapse in exposed women and unexposed women to fertility preservation (*n* = 534) by adjustment in smaller calendar periods to determine an effect of treatment recommendation with adjuvant trastuzumab after Her2 screening introduced in 2005 in the Stockholm healthcare region

Fertility preservation	Model 1^a^ IRR (95% CI)	Model 2^b^ IRR (95% CI)	Model 3^c^ IRR (95% CI)	Model 4^d^ IRR (95% CI)
No Fertility preservation that does not require hormonal stimulation Fertility preservation that requires hormonal stimulation	1.00 (reference)* 0.79 (0.39–1.59) 0.55 (0.31–0.98)	1.00 (reference)* 0.85 (0.42–1.74) 0.62 (0.35–1.09)	1.00 (reference)* 0.79 (0.38–1.61) 0.64 (0.36–1.15)	1.00 (reference)* 0.87 (0.43–1.77) 0.63 (0.36–1.12)
Period of diagnosis				
1997–2000 2001–2004 2005–2008 2009–2013	1.00 (reference) 0.86 (0.55–1.34) 0.44 (0.26–0.72) 0.32 (0.16–0.62)	1.00 (reference) 0.80 (0.51–1.25) 0.41 (0.25–0.69) 0.30 (0.15–0.59)	1.00 (reference) 0.78 (0.50–1.23) 0.39 (0.23–0.65) 0.26 (0.13–0.52)	1.00 (reference) 0.79 (0.50–1.24) 0.42 (0.25–0.70) 0.31 (0.16–0.61)
Tumor size				
T0 TIS I (≤ 20 mm) II (21–50 mm) III (> 50 mm)		0.18 (0.02–1.32) – 1.00 (reference) 1.47 (1.00–2.17) 1.32 (0.73–2.38)	0.18 (0.02–1.30) – 1.00 (reference) 1.19 (0.77–1.84) 0.80 (0.39–1.61)	0.17 (0.02–1.25) – 1.00 (reference) 1.53 (1.02–2.27) 1.39 (0.76–2.55)
Lymph nodes				
0 1–3 > 3		1.00 (reference) 1.70 (1.12–2.59) 2.62 (1.66–4.12)	1.00 (reference) 1.70 (1.12–2.60) 2.43 (1.52–3.88)	1.00 (reference) 2.00 (1.26–3.18) 2.91 (1.73–4.90)
Receptors				
ER+ ER−			1.00 (reference) 1.03 (0.69–1.52)	1.00 (reference) 1.16 (0.77–1.74)
Neoadjuvant treatment				
No Yes			1.00 (Reference) 2.01 (1.22–3.29)	–
Chemotherapy				
No Yes				1.00 (reference) 0.65 (0.37–1.14)

^*CI* confidence interval, *IRR* incidence rate ratio, *ER* estrogen receptor^

^a^Adjusted for age at diagnosis (using a restricted cubic spline with 4 degrees of freedom) and the calendar period of the diagnosis

^b^Further adjusted for tumor size and the number of involved lymph nodes

^c^Further adjusted for estrogen receptor status and neoadjuvant treatment

^d^Adjusted for chemotherapy treatment (pre- or postoperative)

**Fig. 2:**
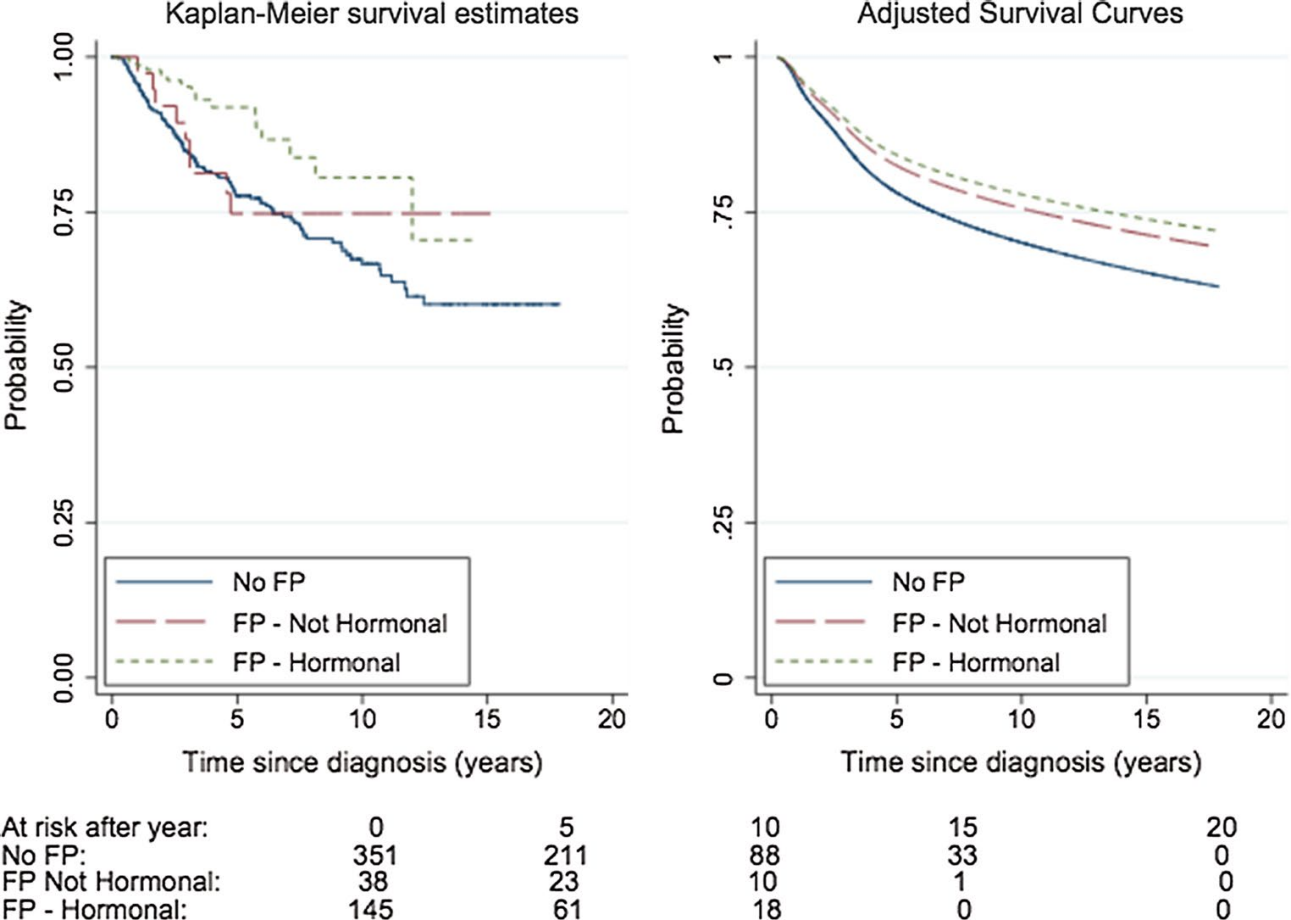
A comparison of relapse-free survival in women who underwent fertility preservation with or without hormonal stimulation and that in women unexposed to fertility preservation (*n* = 534). FP: fertility preservation, Left panel: Kaplan–Meier estimates of relapse-free survival, Right panel: Survival curves adjusted for age and calendar period of diagnosis, tumor size, number of involved lymph nodes, estrogen receptor status, and neoadjuvant treatment estimated using a flexible parametric survival model. The number of women under follow-up at intervals beneath the x-axis is presented

A statistically significant difference was not found between the two groups of exposed and unexposed women using Kaplan–Meier analysis. The survival pattern in the adjusted survival curve was similar to that reflected by the Kaplan–Meier estimates (Fig. 2).

## Discussion

It was shown in this matched cohort study that the performance of procedures in women with breast cancer for which hormonal stimulation was required was not associated with a higher rate of breast cancer relapse, regardless of tumor size, the number of involved lymph nodes, estrogen receptor status, and chemotherapy treatment (pre- or postoperatively).

In terms of clinical implications, based on this study, it can be concluded that controlled hormonal stimulation over a two-week period, needed for the collection of mature oocytes, is unlikely to result in increased breast cancer recurrence risk. Hence, this fertility preservation option can also be offered to women with breast cancer. Our results are relevant and of profound clinical importance as women with breast cancer are predominantly counseled against methods of fertility preservation for which hormonal stimulation is required, despite being clinically established and recognized to be of high efficacy. Nevertheless, methods of reduced efficacy or those still under development can still be presented as suitable options with which to dispel fears about the possibility of hormonal change being induced [4, 17, 24]. It should be noted that before the introduction of tamoxifen as standard adjuvant endocrine therapy for breast cancer, hormonal treatment (including estrogen at high doses and androgen therapy) was used to induce tumor regression in advanced breast cancer cases. The available data collected in a few retrospective studies and one randomized trial (on diethylstilbestrol versus tamoxifen) do not currently support fears of an ensuing negative impact on breast cancer disease progression as a result of an increase in estradiol levels having been effected for a limited period [25–27].

Oncological outcome, regarding relapse rates in women with breast cancer who have undergone fertility preservation according to a common standardized stimulation protocol, was also covered in our study. It is noteworthy that the number of women undergoing hormonal stimulation for fertility preservation is currently increasing. In our cohort, 107 women elected to receive such treatment from 2008 to 2013, representing an increment of ≥ 200% over the previous period. Relapses occurred less frequently in women receiving hormonal stimulation than in those who did not or in controls who were not exposed to fertility preservation (Table 1). The IRR was also consistently low in all the adjusted models for women who received hormonal stimulation (Table 2). Although approximately half of these women presented with estrogen-negative breast cancer, our data are reassuring as they do not indicate that the hormonal stimulation required for fertility preservation was associated with an increased risk of relapse, independent of estrogen receptor status. The introduction in 2010 of the modified protocol, with the addition of letrozole [16], may have potentially increased the safety of hormonal stimulation. This is indicated by the recorded relapse figures (within the lowest range) for women who underwent hormonal stimulation from 2008 to 2013 (Table 1).

We were unable to perform a subset analysis of a restricted cohort to specifically evaluate the safety of stimulation protocols using letrozole in this study owing to sparse data and the limited follow-up of women diagnosed with breast cancer in 2010 or later. The safety of the addition of letrozole in that clinical context was reported in two previous studies, both from a single cohort [14, 15]. However, the selection bias in that cohort was plausible as significantly less frequent lymph node involvement was observed in women who were treated with letrozole than that in the control group (*p* value = 0.020) [15].

Our study design took the form of a large population-based study, with complete coverage and long-term follow-up performed to specifically examine the effect of fertility preservation by any currently available method on women with breast cancer. Conducting a randomized study is not currently feasible as fertility preservation depends upon patient election.

Strengths of our study include the performance of fertility preservation via a regional healthcare program with full population coverage and equal access to care, as well as the unique method of utilizing the Swedish Cancer Registry for the corresponding healthcare region to match exposed women with appropriate comparators who had received similar health care for breast cancer treatment within the same calendar period. As access to counseling and the performance of fertility preservation are not restricted in Sweden and are provided to all citizens, financial status (a potential selection bias in the current study) was not a limiting factor in our case but might be so elsewhere. Although similarities in the clinical presentation of breast cancer between the exposed and unexposed study cohorts were observed (Table 1) and our data do not support the hypothesis that women who receive fertility preservation have less advanced disease than the matched controls, additional lifestyle aspects did not constitute part of the study criteria and therefore were not identified and included. Thus, residual confounding as a result of lifestyle differences cannot be ruled out. The reason for not including Her2 status or grade as potential confounders was that these factors did not impact upon the decision-making process regarding whether or not to proceed with fertility preservation treatment.

Knowledge of the risks associated with fertility preservation is limited, particularly because the field of fertility preservation is relatively young. This makes appropriate counseling difficult [28–30]. The practice of fertility preservation for women with breast cancer, irrespective of the need for hormonal stimulation, is supported by our study findings.

## Conclusions

We found in this matched cohort study on women with breast cancer that using gonadotropin stimulation, with or without letrozole, for fertility preservation purposes, is unlikely to result in a substantially increased risk of cancer recurrence. Although the limited sample size may weaken an interpretation of the findings, the high coverage rate of this population-based study supports the premise that the practice of fertility preservation is safe in young women with breast cancer. Further research, including a longer-term follow-up of a large cohort, is needed to confirm these findings.
